# Identification and Removal of Pollen Spectral Interference in the Classification of Hazardous Substances Based on Excitation Emission Matrix Fluorescence Spectroscopy

**DOI:** 10.3390/molecules29133132

**Published:** 2024-07-01

**Authors:** Pengjie Zhang, Bin Du, Jiwei Xu, Jiang Wang, Zhiwei Liu, Bing Liu, Fanhua Meng, Zhaoyang Tong

**Affiliations:** State Key Laboratory of NBC Protection for Civilian, Beijing 102205, China; zpjbit@163.com (P.Z.); dubin51979@163.com (B.D.); xujw14@mail.ustc.edu.cn (J.X.); roverman@163.com (J.W.); liuzhw07@lzu.edu.cn (Z.L.); lbfhyjy@sohu.com (B.L.); mfh027@163.com (F.M.)

**Keywords:** pollen interference, random forest, toxin, pathogenic bacteria, excitation emission matrix

## Abstract

Sensitively detecting hazardous and suspected bioaerosols is crucial for safeguarding public health. The potential impact of pollen on identifying bacterial species through fluorescence spectra should not be overlooked. Before the analysis, the spectrum underwent preprocessing steps, including normalization, multivariate scattering correction, and Savitzky–Golay smoothing. Additionally, the spectrum was transformed using difference, standard normal variable, and fast Fourier transform techniques. A random forest algorithm was employed for the classification and identification of 31 different types of samples. The fast Fourier transform improved the classification accuracy of the sample excitation–emission matrix fluorescence spectrum data by 9.2%, resulting in an accuracy of 89.24%. The harmful substances, including *Staphylococcus aureus*, ricin, beta-bungarotoxin, and Staphylococcal enterotoxin B, were clearly distinguished. The spectral data transformation and classification algorithm effectively eliminated the interference of pollen on other components. Furthermore, a classification and recognition model based on spectral feature transformation was established, demonstrating excellent application potential in detecting hazardous substances and protecting public health. This study provided a solid foundation for the application of rapid detection methods for harmful bioaerosols.

## 1. Introduction

Bioaerosol sources include both human and natural sources, with various aerosols being produced throughout the life cycle of plants and animals [1,2]. Hazardous substances in bioaerosols include pathogenic bacteria and biotoxins, such as *Staphylococcus aureus* (*S. aureus*), ricin, abrin, and beta-bungarotoxin (BGT), which present a significant risk to the health of the general public [3,4]. Among these sources, plant pollen is a common component of bioaerosols due to its wide distribution and similar composition, which can complicate the monitoring of bioaerosols [5]. Additionally, pollen can travel long distances in the air, posing a potential threat to humans due to its own presence and the pathogenic particles it may carry [6]. The rise in suspicious airborne particles has resulted in increased mortality rates. The swift detection of noxious and detrimental aerosols is imperative for safeguarding human well-being. Fluorescence-spectrum-based monitoring technology was primarily focused on meeting the future needs for monitoring and early warning of harmful biological aerosols, making it the primary focus of bioaerosol monitoring. It should be noted that the identification of bacteria and other substances may be influenced by environmental factors [7]. Additionally, the fluorescence spectrum of pollen closely resembled that of biological source components, thus presenting a significant interference challenge due to pollen’s strong emission characteristics [8]. However, there is a lack of systematic studies on the impact of pollen on the classification of biogenic components. Therefore, it is crucial to investigate the influence of pollen on the classification and recognition of other components and develop a method for classifying and recognizing biogenic components that can eliminate the interference from pollen.

Three-dimensional fluorescence spectra, also known as excitation–emission matrix fluorescence spectroscopy (EEM), can provide both excitation and emission wavelength information [9,10]. It has been reported that EEM is utilized for the classification of samples belonging to different subclasses within the same species [11,12,13]. Relatively little research has been conducted on the classification of various types of samples, such as pollen, bacteria, and proteins. However, the fundamental reason pollen spectral characteristics affect the classification and recognition of bacterial spectral characteristics was unclear. In previous studies, chemometric methods and machine learning algorithms were utilized for the processing and recognition of spectral data [14,15,16]. These techniques include multivariate scattering correction (MSC), Savitzky–Golay smoothing (SG), standard normal variable transformation (SNV), fast Fourier transform (FFT), partial least squares discriminant analysis (PLS-DA), and the random forest algorithm (RF). The continuous development of machine learning algorithms brought new opportunities for research in spectral classification and recognition technology, providing a valuable analytical tool for revealing the influence of pollen spectral characteristics [17]. With the increasing variety of sample types, it is crucial to establish a more comprehensive biogenic component profile database [18]. This will lay the foundation for the future development of online monitoring technology. The collection method of aerosol particles has been utilized for classifying static fluorescence spectra in laboratory settings [19,20,21]. This collection method, when combined with a classification model, allows for the extraction and classification of spectral features to identify unknown biological particles [22]. The performance of the classification model is closely related to spectral characteristics and improves as more spectral characteristics are inputted into the model [23]. However, simply adding spectral features does not lead to continuous improvement in the model’s performance. Therefore, a new approach that involves the fusion and application of different spectral information is necessary for effectively analyzing spectral characteristics.

In this study, we investigated the relationship between the spectral characteristics of pollen and other substances. The spectral characteristics were extracted using algorithms, and a successful classification was achieved. Our research focused on reducing or eliminating the interference of pollen on bacterial classification recognition. The development of new monitoring technology requires a model with good classification performance for various biological particles [24,25]. Three types of bacteria were used in the laboratory to replace pathogenic bacteria and establish a bacterial detection model based on the fluorescence spectrum. Static fluorescence spectra of all samples were collected and pre-treated. Then, the spectral data were processed through feature extraction and classification algorithms. The transformation of spectral features improved the model’s classification ability. This study aimed to explore a method for removing pollen spectral feature interference using machine learning. Fluorescence spectral signatures were utilized to eliminate the interference between pollen and bacteria, as well as to classify them [26]. This study aimed to classify the fluorescence spectra of pollen, bacteria, toxins, proteins, amino acids, and other samples for the first time. Additionally, it sought to mitigate the effects of pollen by altering spectral characteristics. This strategy may hold great potential for application in mixed spectrum classification and target recognition under interference conditions, laying a theoretical foundation for the future development of real-time monitoring and early warning devices for biological warfare agents based on this model.

## 2. Results

### 2.1. Classification and Recognition of the Original Spectrum

**Figure 1:**
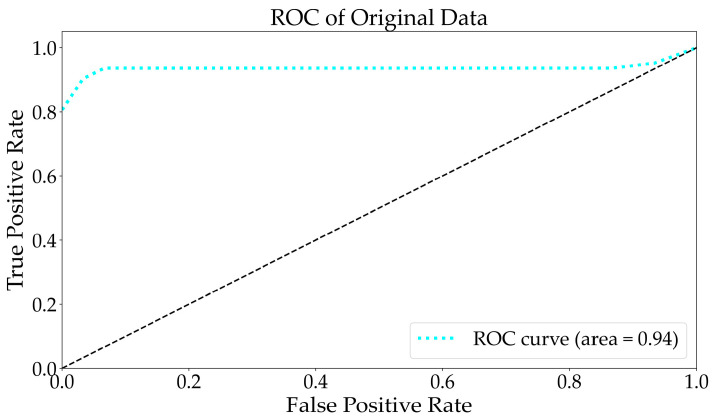
The ROC curve analysis for multiple classification tasks using the original EEM spectral data. The black dashed represents the identity.

**Figure 2:**
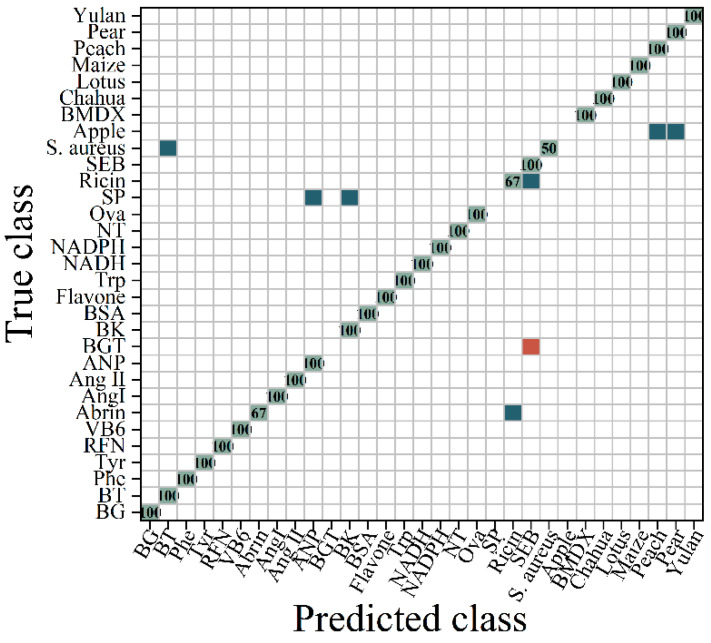
RF classification confusion matrix of EEM spectral data. (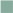 represents the accurate prediction of the sample, with the number indicating the proportion of correctly predicted samples within the total samples in the class; 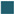 indicates that a portion of the samples were predicted to belong to another category; 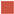 signifies that the samples were predicted to belong to a different category).

After completing the model prediction, a probability matrix was formed by calculating the probability of testing each sample under each classification. From this matrix, the score of the test sample under each category could be obtained, thus forming a score matrix similar to a binary classification. Following the method for drawing receiver operating characteristic (ROC) curves in a binary classification, the false positive case rate (FPR) and true positive case rate (TPR) could be calculated under each threshold based on the aforementioned matrix in order to draw an ROC curve. One ROC curve could be drawn for each category, resulting in a total of n ROC curves. Finally, averaging these n ROC curves yielded the final ROC curve. As illustrated in Figure 1, the area under the curve (AUC) was also provided (area = 0.94). The original spectra were classified and are presented in Figure 2. The RF algorithm divided the original spectra into 28 categories, which could not distinguish between bacteria, viruses, pollen, and polypeptides. This might have been due to the similar spectral characteristics within samples of the same class, with no discrimination error observed between the samples of different classes.

Random forests were able to accurately predict four out of the five categories of pollen biomes, which were arid, mountainous, tropical, and subtropical closed and open systems [27]. Fourier transform infrared spectroscopy (FTIR) was employed to classify the pollen, resulting in a classification accuracy of 75% for five different pollen samples [28]. Limited research has been conducted on the use of EEM for bacterial classification [29]. The EEM spectrum was utilized to accurately classify two out of three bacteria and seven out of eight pollens, achieving an accuracy rate of 81.72%. It is evident that in comparison with Raman spectroscopy and infrared spectroscopy, three-dimensional fluorescence spectroscopy may provide a more accurate description of sample characteristics. Consequently, the classification ability of pollen and bacteria samples was enhanced. The results indicate that the EEM spectra were specific.

### 2.2. Spectral Transformation and Classification

#### 2.2.1. Spectral Transformation

**Figure 3:**
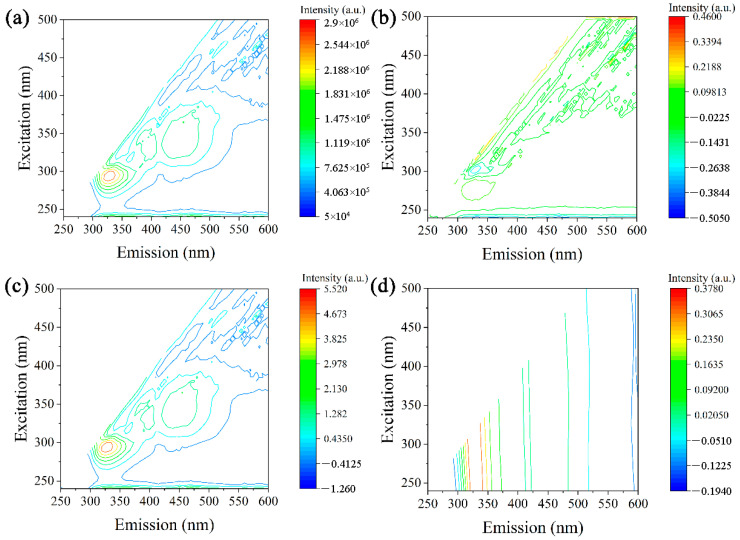
Fluorescence contour map of *Bacillus atrophaeus*: (**a**) original, (**b**) D1 transformation, (**c**) SNV transformation, and (**d**) FFT transformation.

As shown in Figure 3, the primary fluorescence region of the original spectrum was within the excitation wavelength range of 275 nm to 300 nm and emission wavelength range of 300 nm to 350 nm. Following the D1 conversion, there was a decrease in the main fluorescence region and fluorescence intensity, while an increase in the fluorescence intensity was observed within the range of 400 nm to 600 nm. The SNV method was applied to the EEM spectrum data. Following the transformations of Bacillus subtilis and Bacillus thuringiensis, the excitation wavelength range of the BG spectrum was 300 nm to 375 nm, with an emission wavelength range of 375 nm to 425 nm. The FFT transformation exhibited significant variation from 350 nm to 600 nm.

**Figure 4:**
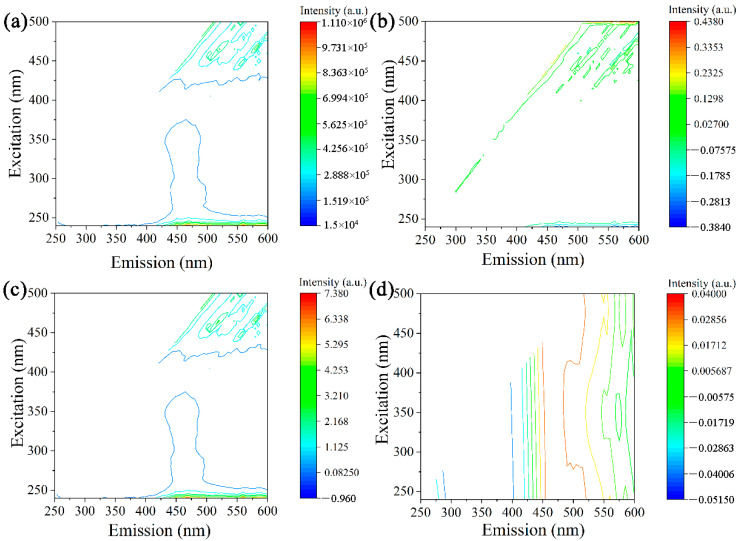
Fluorescence contour map of peach: (**a**) original, (**b**) D1 transformation, (**c**) SNV transformation, and (**d**) FFT transformation.

As depicted in Figure 4, the fluorescence intensity of the pollen spectrum was notably weak, and the main fluorescence region was not visible after the first-order difference transformation (D1). After the D1 transformation, the spectrum of the peach blossom powder was significantly different from the original spectrum. Following the SNV transformation, there was little change in the main fluorescence region, with a shift in excitation wavelength of 450–500 nm. The FFT transformation exhibited significant variation from 400 nm to 600 nm. The region of stronger fluorescence was within the 450–525 nm range, which differed from the other transformed data.

#### 2.2.2. Transformation Spectra’s Classification Performance

After the transformation of spectral data, the classification performance of RF is presented in Table 1. It is evident from the table that all three transformation methods enhanced the spectral classification effect, with FFT being the most effective. The out-of-bag (OOB) error estimate indicated that the classification performance of the FFT-transformed data was superior to both the D1 and original data.

**Figure 5:**
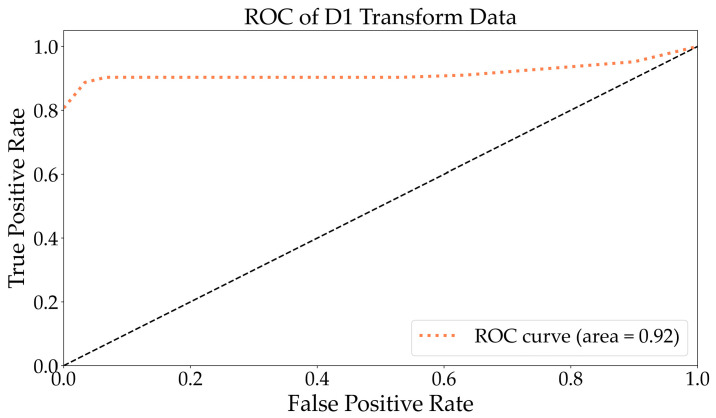
The ROC curve analysis for multiple classification tasks using the D1-transformed data. The black dashed represents the identity.

**Figure 6:**
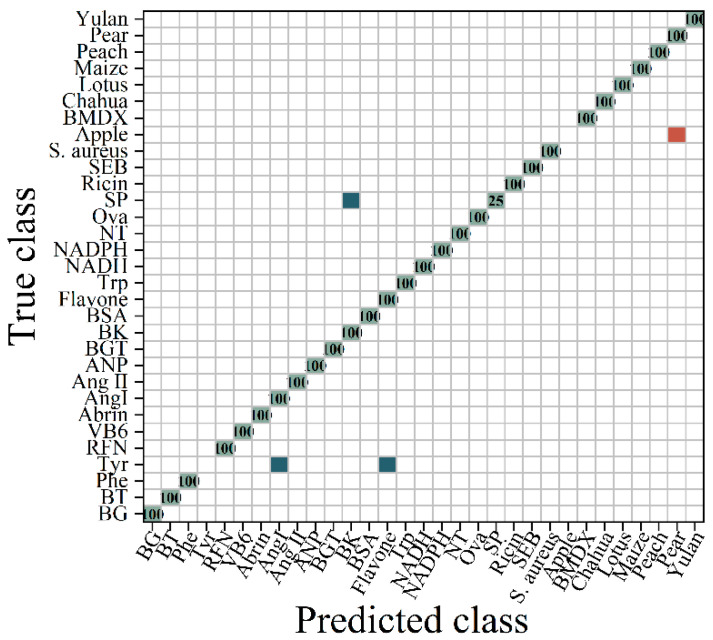
The confusion matrix diagram of the RF classification for the D1-transformed spectra. (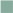 represents the accurate prediction of the sample, with the number indicating the proportion of correctly predicted samples within the total samples in the class; 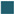 indicates that a portion of the samples were predicted to belong to another category; 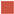 signifies that the samples were predicted to belong to a different category).

As depicted in Figure 5, the ROC to multi-class following D1 exhibited an AUC of 0.92, which was slightly lower than that of the original data. The ROC curve demonstrates the average of 31 samples. As illustrated in Figure 6, the RF algorithm divided the D1 transform spectrum into 29 categories and was capable of accurately distinguishing between bacteria and viruses. However, it incorrectly identified apple pollen as pear pollen and struggled to differentiate Tyr from polypeptides and flavonoids. This indicates that the D1 differential treatment effectively highlighted the fluorescence spectral characteristics of bacteria and viruses. The accuracy increased by 7.89% compared with the original spectra.

**Figure 7:**
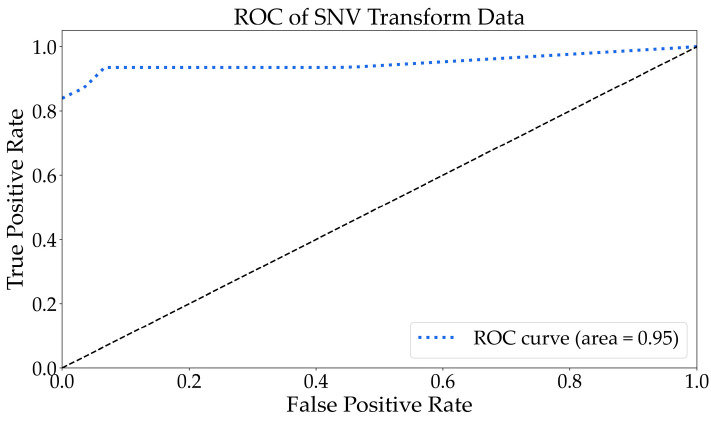
The ROC curve analysis for multiple classification tasks using the SNV-transformed data. The black dashed represents the identity.

**Figure 8:**
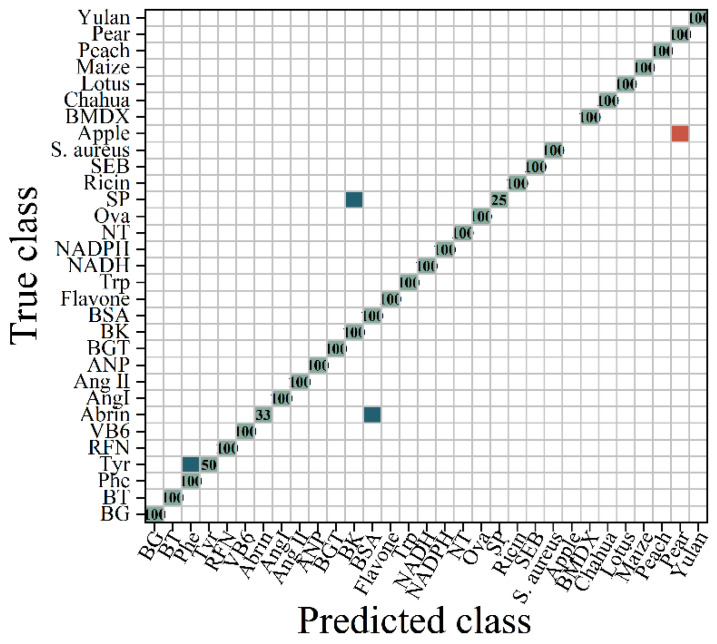
The confusion matrix diagram of the RF classification for the SNV-transformed spectra. (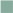 represents the accurate prediction of the sample, with the number indicating the proportion of correctly predicted samples within the total samples in the class; 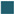 indicates that a portion of the samples were predicted to belong to another category; 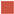 signifies that the samples were predicted to belong to a different category).

As depicted in Figure 7, the ROC to multi-class following the SNV transformation exhibited an area under the curve of 0.95, which was higher than that of the original data. The AUC was closer to 1, indicating superior classification results. Therefore, it suggests that the classification performance was excellent. As shown in Figure 8, the RF algorithm categorized the SNV-transformed spectrum into 30 groups. In comparison with the original spectrum, bacteria were clearly distinguished, and abrin was identified as BSA, while amino acids were less distinguishable. This indicated that the SNV transformation method optimized the discriminative features of bacteria and enhanced the classification recognition of bacteria. The accuracy increased by 7.89% compared with the original spectral data.

**Figure 9:**
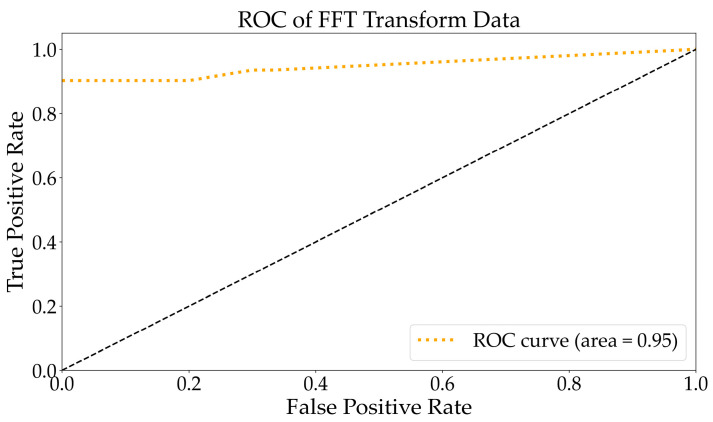
The ROC curve analysis for the multiple classification tasks using the FFT-transformed data. The black dashed represents the identity.

**Figure 10:**
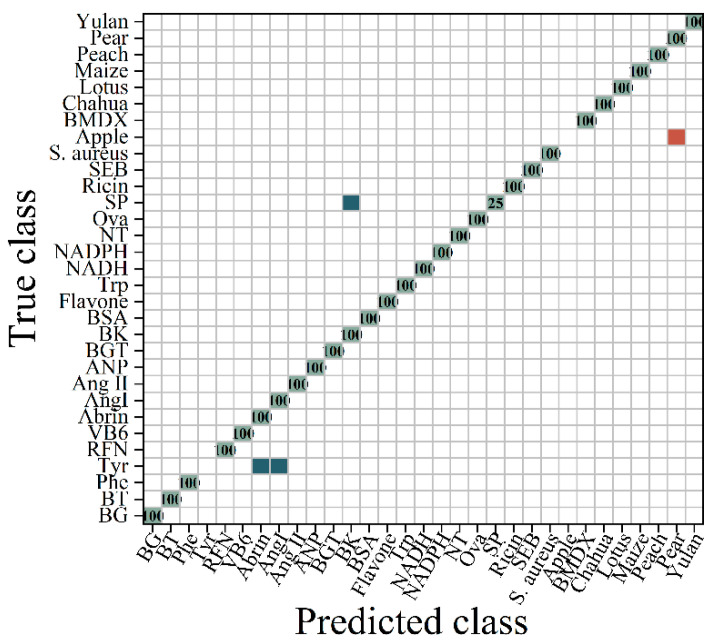
The confusion matrix diagram of the RF classification for the FFT-transformed spectra. (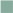 represents the accurate prediction of the sample, with the number indicating the proportion of correctly predicted samples within the total samples in the class; 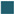 indicates that a portion of the samples were predicted to belong to another category; 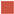 signifies that the samples were predicted to belong to a different category).

As depicted in Figure 9, the ROC to multi-class following the FFT transformation exhibited an area under the curve of 0.95, which was higher than that of the original data. The ROC curve differed from the SNV transform, but the AUC was equivalent to the SNV transform. This indicates that certain samples were categorized differently within the two datasets. Based on Figure 8 and Figure 10, it can be observed that the classification results for abrin and Tyr were dissimilar. Figure 10 illustrates the division of the FFT-transformed spectra into 29 categories by the RF algorithm. Bacillus subtilis, Bacillus thuringiensis, *Staphylococcus aureus*, ricin, beta-bungarotoxin, and staphylococcal enterotoxin B were accurately distinguished. Amino acids posed a challenge in distinguishing them from toxins and peptides, with Tyr being identified as abrin and Ang I. After undergoing the FFT transformation, the data were observed to effectively distinguish between abrin and BSA. This suggests that their characteristics were valuable for identifying similar components, indicating the effectiveness of the transformation. The classification accuracy of the sample fluorescence spectrum data was enhanced by 9.2% to reach 89.24%.

## 3. Discussion

This study demonstrated that bacterial fluorescence spectra analysis could effectively categorize bacteria based on their characteristics using samples such as bacteria, bioactive substances, and pollen. Common biological agent mimics include BG, BT, and Ova. In addition to the aforementioned two bacteria, *S. aureus* was also included in the study. The experimental results suggest that the bacteria may be incomplete in a natural environment, leading to the exposure of certain proteins and amino acids. Therefore, BSA, Ova, and three amino acids were added to the sample. Additionally, NADH and NADPH are crucial bioactive components involved in cell metabolism. As a result, this study aimed to simulate the complex components of atmospheric aerosols as accurately as possible, including bacterial components and major disturbing factors. Thus far, a relatively simple and targeted portfolio of microatmospheric aerosols was established.

Fluorescence spectral features were utilized for substance classification [30,31]. The sample being tested may exist in solid or liquid form. This study primarily focused on the analysis of common solid samples. This study focused on economically and rapidly analyzing dry powder substances with sufficient reproducibility. After collecting spectral data information from all samples, various machine learning algorithms were employed to conduct in-depth research on the modeling feasibility of each spectrum.

The results of the analysis based on the fluorescence spectra demonstrated that the described analytical method could effectively classify bacteria and reference substances, identify their similarities, and rapidly analyze the structural features of a small number of samples. Additionally, the coefficient of determination (R^2^), root-mean-square error (RMSE), predictive correlation coefficient, and determination coefficient were taken into consideration [32]. The accuracy, confusion matrix, and receiver operating characteristic curve were also utilized for evaluating the classification performance [33]. The results indicated that the transformed spectra demonstrated superior performance when using the RF model (Table 2). Current research on the classification, biological composition, and pollen of bacteria is currently limited in scope. Therefore, this study aimed to address this knowledge gap by attempting to combine two spectral features to improve the recognition performance of the model under pollen interference.

**Figure 11:**
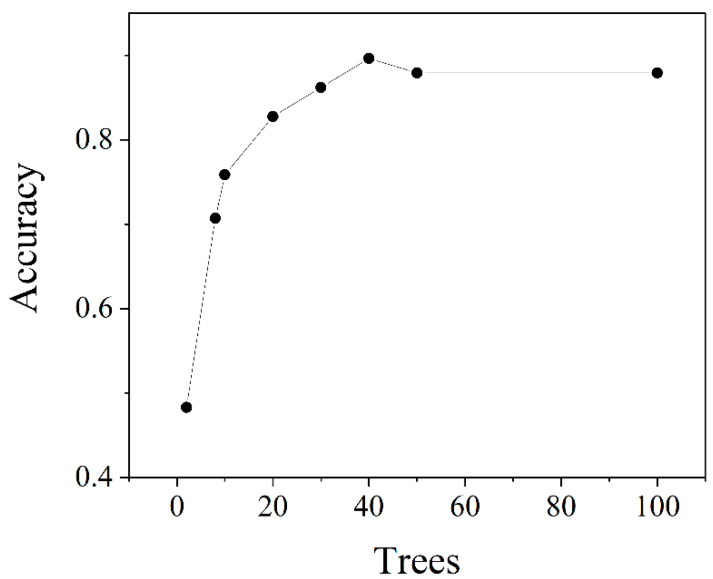
The accuracy–trees relationship of the RF classification EEM spectral data.

The predicting performance of the RF method was affected by the N tree. The N tree value was determined by the spectral classification results. Therefore, proper adjustments were made to the N factor to enhance the recognition performance. As shown in Figure 11, with the increase in n, the accuracy rate gradually increased. The highest accuracy was achieved when n equaled 40. However, as n continued to rise beyond this value, the accuracy decreased. Therefore, in all the experiments below, the value of N was selected as 40.

## 4. Materials and Methods

**Figure 12:**
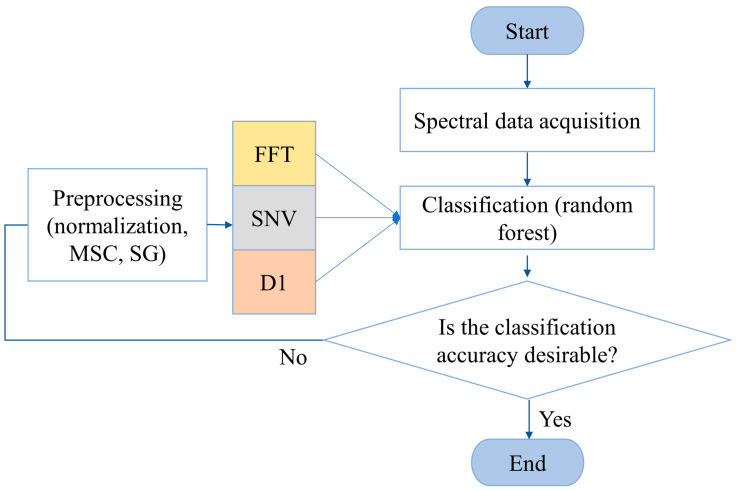
Flowchart of the proposed method.

The proposed algorithm included 5 steps (Figure 12). The first step involved acquiring the EEM spectra, which were then tested on 31 different samples. The next step involved algorithmically classifying the raw data. The classification method utilized was the RF algorithm. If the classification accuracy was desirable, the process was terminated. Otherwise, the process proceeded to the next step. The spectral data were preprocessed, which included normalization, an MSC, and SG. Subsequently, the preprocessed data underwent D1, followed by SNV and FFT processing. Finally, the processed data were categorized using an RF model.

### 4.1. Materials and Biological Samples

Thirty-one samples were selected for the fluorescence spectrum collection in this study. *Bacillus atrophaeus* (BG) and *Bacillus thuringiensis* (BT) were cultivated and provided by our laboratory. *Staphylococcus aureus* (*S. aureus*) was purchased from the BeNa Culture Collection (Beijing, China) and cultivated in the laboratory. The remaining samples were purchased commercially (see Table 3). The EEM spectra were collected using an FLS1000 spectrometer (Edinburgh Instruments Ltd., Livingston, UK) in the 200 nm–800 nm range with a 5 nm step size. A total of five spectra were recorded for each sample. Ultrapure water was used in the experiment (MILLIPORE, Billerica, MA, USA). Bacteria in the logarithmic phase were subjected to a vacuum freeze-drying treatment, and the remaining reagents were used without further purification.

The pollen species studied were obtained from apple, *Syringa reticulata var. amurensis* (BMDX), *Camellia japonica* (chahua), lotus, maize, peach, pear, and *Magnolia denudata* (yulan) flowers. Both riboflavin (RFN) and vitamin B6 (VB6) are essential vitamins. Both nicotinamide adenine dinucleotide (NADH) and nicotinamide adenine dinucleotide phosphate (NADPH) served as coenzymes. Atrial natriuretic peptide (ANP), angiotensin I (Ang I), angiotensin II (Ang II), bradykinin (BK), substance p (SP), and neurotensin (NT) are all polypeptides. Tryptophan (Trp), tyrosine (Tyr), and phenylalanine (Phe) are three essential amino acids. Bovine serum albumin (BSA) and ovalbumin (Ova) are two commonly found proteins in the academic literature.

### 4.2. The Excitation Emission Matrix Fluorescence Spectral Measurements

The EEM spectra of biological source components were measured in a front-face configuration using the FLS1000 steady-state/transient fluorescence spectrometer (Edinburgh Instruments, Livingston, UK), which was equipped with a 450 W ozone-free xenon arc lamp. The instrument featured a PMT-900 detector and operated in a cooling environment at minus 20 °C, covering a spectral range of 185 nm to 900 nm (recommended range of 200 nm to 870 nm).

Sample preparation: An appropriate amount of sample powder was taken and placed on the solid fluorescent sample pool using a medicine spoon. We ensured that the powder was slightly higher than the sample pool, and then covered it with a lid and gently pressed to create a smooth surface. The medicine spoon was used to remove any excess sample outside of the pool. Once the sample preparation was complete, it was placed on the sample rack in the instrument for the spectrum test to begin.

Test conditions: The excitation wavelength was selected to be in the range of 240 nm to 500 nm, with a step size of 5 nm and a slit width of 1.5 nm. The emission wavelength ranged from 250 nm to 600 nm, with a step size of 5 nm and a slit width of 1.5 nm. The voltage intensity was set at 400 V, and the integration time was 0.1 s. All experiments were conducted at room temperature.

To facilitate the subsequent data processing, the EEM spectrum data measured by the fluorescence spectrometer were converted into CSV format, resulting in a two-dimensional matrix of spectral data. The processed two-dimensional spectral data was further transformed into one-dimensional data by connecting adjacent rows end to end in the direction of the emitted wavelength. This conversion allowed for easier handling and analysis of the spectral information.

### 4.3. Data Treatment

The selected spectra preprocessing methods, which were written in Python (PyCharm Community Edition 2021.3.1), included spectral normalization, scattering correction, and smoothing. The spectra were treated with the MSC and SG to reduce the noise level [34,35]. The parameters of the SG filter were optimized as follows: the window length was set to 5, the polynomial order was chosen as 3, and the mode was specified as nearest.

The D1 transformation calculates the difference between two consecutive adjacent items in discrete data. When the independent variable changes from *x* to *x* + 1, the change in function Δ*y* is called the first-order difference of *y* at *x*, as in Equation (1):

(1) $$\Delta y_{x}=y_{x+1}-y_{x}$$

The *SNV* transformation calculates the standard deviation of each sample to correct the spectrum [36], as in Equation (2):

(2) $$X_{SNV}=\frac{\bar{x}-x}{\sqrt{\frac{1}{m-1}\sum_{k=1}^{m}{\left(x_{k}-\bar{x}\right)}^{2}}}$$

where $\bar{x}=\sum_{k=1}^{m}x_{k}/m$; *m* is the number of wavelengths; and k=1,2,…,m.

The FFT is a rapid computational method for calculating the discrete Fourier transform [37]. For an *N*-point sequence, as in Equation (3):

(3) $$X(k)=\sum_{r=0}^{\frac{N}{2}-1}\left[x(n)+x\left(n+\frac{N}{2}\right)\right]W_{N}^{2rn}+W_{N}^{n}\sum_{r=0}^{\frac{N}{2}-1}\left[x(n)-x\left(n+\frac{N}{2}\right)\right]W_{N}^{2rn}$$

where $x{\left[n\right]}_{0\leq n\leq N}$; $W_{N}=e^{-j\frac{2\pi }{N}}$; k=0,1…,N−1; and r=0,1…,N/2−1.

The RF algorithm was applied to extract spectral features for the classification of biological components [38]. *Gini* (*D*) reflects the probability that two randomly selected samples from dataset *D* belong to different categories. In attribute set *A*, the attribute with the smallest Gini coefficient is chosen as the optimal partition attribute. *H^OOB^*(*x*) represents an *OOB* prediction for sample *x* [39]. The formulas are as follows:

(4) $$\begin{array}{c} Gini(A,a)=\sum_{v=1}^{V}\frac{\left|D^{v}\right|}{\left|D\right|}Gini\left(D^{v}\right)\\ a_{*}=\underset{a\in A}{\arg\;\min}\;Gini(D,a) \end{array}$$

(5) $$H^{OOB}(x)=\underset{y\in y}{\arg\;\max}\sum_{t=1}^{T}I\left(h_{t}(x)=y\right)\cdot I\left(x\notin D_{t}\right)$$

### 4.4. Performance Evaluation Metrics

The model’s performance was evaluated by employing the R^2^ and RMSE. The confusion matrix (CM) accurately depicts the predictive results of a classifier. True positive (TP, positive samples correctly classified), false negative (FN, negative samples incorrectly classified), false positive (FP, positive samples incorrectly classified), and true negative (TN, negative samples correctly classified) are employed to evaluate the performance of a classifier [40]. The accuracy metric represents the ratio of accurate predictions to the total number of predictions made. The precision is determined by dividing the count of accurately predicted positive instances by the total count of predicted positive class values. The recall is computed as the ratio of true positive predictions to the total number of actual positive values in the test dataset. The F1-score represents the harmonic mean of the precision and recall ratios. The equations for all metrics are shown in Table 4.

## 5. Conclusions

In this study, the EEM spectra were preprocessed and transformed. The RF algo-rithm was employed to extract and classify the features of the biological samples, such as bacteria and pollen. After the transformation of fluorescence spectral data, the classification performance was enhanced. The data processed by D1 and SNV achieved an accuracy rate of 88.17% in the classification. The accuracy increased by 7.89% compared with the original data. The RF model achieved the highest classification accuracy of 89.24% for the FFT-transformed data, highlighting its exceptional performance in accurately classifying the data. The outcome was that bacteria, proteins, toxins, and pollen were effectively separated, regardless of the impact of pollen on the classification. The spectral characteristics of the EEM spectra were effectively classified using the RF method. Multiple samples were successfully distinguished, including bacteria, toxins, proteins, vitamins, coenzymes, and pollen. This classification model successfully categorized the spectra of 31 biological samples and effectively eliminated pollen interference. This approach established a solid foundation for the development of online monitoring technology utilizing fluorescence spectra in future research.

## Figures and Tables

**Table 1:** The classification metric results from the original fluorescence spectrum and transformed data.

Transform	Accuracy	Precision	Recall	F1-Score	OOB Error
N	0.8172	0.7897	0.8658	0.8016	0.6613
D1	0.8817	0.8590	0.9006	0.8548	0.7581
SNV	0.8817	0.8909	0.9058	0.8635	0.7903
FFT	0.8924	0.8751	0.9113	0.8732	0.7903

N means the data was not transformed.

**Table 2:** The determination coefficient and root-mean-square error of random forest classification of the original fluorescence spectrum and transformed data.

Evaluation Index	N	D1	SNV	FFT
R^2^	0.8001	0.9348	0.9407	0.9544
RMSE	4.017	2.293	2.187	1.917

**Table 3:** The main information of the reagents for the fluorescence spectroscopy study.

Sample	Company	Purity
Atrial natriuretic peptide	APExBIO (Houston, TX, USA)	95%
Angiotensin I	APExBIO	96%
Angiotensin II	APExBIO	96%
Bradykinin	APExBIO	99%
Substance p	APExBIO	99%
Neurotensin	APExBIO	98%
Bovine serum albumin	Solarbio (Beijing, China)	97%
Ovalbumin	Solarbio	Biotechnology grade
Nicotinamide adenine dinucleotide	Aladdin (Shanghai, China)	99%
Flavone	Aladdin	98%
Tryptophan	Aladdin	99%
Tyrosine	Aladdin	99%
Phenylalanine	Aladdin	99%
Vitamin B6	Aladdin	98%
Nicotinamide adenine dinucleotide phosphate	Macklin (Shanghai, China)	96%
Riboflavin	Macklin	98%
Abrin	Beijing H&P Biomedical Institute (Beijing, China)	High purity
Ricin	Beijing H&P Biomedical Institute	High purity
Staphylococcal enterotoxin B	Beijing H&P Biomedical Institute	High purity
*β*-bungarotoxin	Beijing H&P Biomedical Institute	High purity
Pollen	Xinzhou Wutai Mountain Bee Industry Company (Xinzhou, China)	-

The symbol “-” denotes the purity of the unspecified material.

**Table 4:** The formulas for evaluating the classification model metrics.

Metrics	Accuracy	Precision	Recall	F1-Score
Equation	$\frac{\mathrm{TP}\;+\;\mathrm{TN}}{\mathrm{TP}\;+\;\mathrm{TN}\;+\;\mathrm{FP}\;+\;\mathrm{FN}}$	$\frac{\mathrm{TP}}{\mathrm{TP}\;+\;\mathrm{FP}}$	$\frac{\mathrm{TP}}{\mathrm{TP}\;+\;\mathrm{FN}}$	$\frac{2\;\times \;\mathrm{Precision}\;\times \;\mathrm{Recall}}{\mathrm{Precision}\;+\;\mathrm{Recall}}$

## Data Availability

The data presented in this study are available in the article.
